# Factors associated with the self-perceived ability of nursing staff to remain working until retirement: a questionnaire survey

**DOI:** 10.1186/s12913-015-1006-x

**Published:** 2015-09-02

**Authors:** Erica E. M. Maurits, Anke J. E. de Veer, Lucas S. van der Hoek, Anneke L. Francke

**Affiliations:** Netherlands Institute for Health Services Research (NIVEL), Utrecht, The Netherlands; Department of Public and Occupational Health, EMGO Institute for Health and Care Research (EMGO+), VU University Medical Center, Amsterdam, The Netherlands

## Abstract

**Background:**

It is important to learn how employers in European countries can prevent nursing staff from changing occupation or taking early retirement in order to counteract expected nursing shortages. However, to date research on nursing staff’s ability to remain working until retirement age has been limited. The purpose of this study was to gain insight into the associations between different job and organisational characteristics, job satisfaction, occupational commitment and the self-perceived ability to continue working in the current line of work until the official retirement age.

**Methods:**

The questionnaire-based, cross-sectional study included 730 nursing staff members employed in Dutch hospitals, nursing homes, organisations for psychiatric care, homes for the elderly, care organisations for disabled people and home care organisations (mean age: 48; 89 % female). Linear and logistic regression analyses and mediation analyses were applied to test hypothesised associations.

**Results:**

Reducing work pressure and increasing appreciation by senior management in particular have positive consequences for nursing staff’s self-perceived ability to continue working until the official retirement age. The job and organisational characteristics of autonomy, work pressure, supportive leadership, educational opportunities, communication within the organisation and appreciation of nursing staff by senior management together have substantial impact on nursing staff’s job satisfaction. Job satisfaction in turn is related to the self-perceived ability to continue working until the retirement age. However, job satisfaction mainly summarises the joint effect of job and organisational characteristics and has no supplementary effect on the self-perceived ability to continue working.

**Conclusion:**

Employers should primarily focus on work pressure and the appreciation of nursing staff by senior management in order to retain nursing staff even as they get older.

**Electronic supplementary material:**

The online version of this article (doi:10.1186/s12913-015-1006-x) contains supplementary material, which is available to authorized users.

## Background

A nursing shortage is expected in the coming years in most countries in the WHO European Region due to increasing care needs. The need for direct patient care has been rising as a result of multiple forces in European societies, such as aging populations, the increasing prevalence of chronic diseases and higher survival rates for seriously ill people. Furthermore, there is a threat of a shortage of nurses due to nursing staff retiring or leaving the profession [1, 2]. Reducing the flow of nursing staff leaving their profession might help to counteract the expected shortages of nursing staff in European countries. This is even more important because we have indications that nursing staff often do not expect to remain working in the nursing profession until retirement age. In the Netherlands, for instance, the age up to which healthcare workers expect to be capable of working in their profession is lower than for other occupational groups. Only craftspeople, industrial workers and farmers expect to have to stop working at a lower age than healthcare workers do [3].

It would thus be of interest to learn how employers can prevent nursing staff members from leaving the profession and retain them for patient care, even as they get older. In addition, it is important for healthcare organisations to gain insight into the job and organisational factors that are related to the self-perceived ability of nursing staff to remain working in their current line of work until the official retirement age. Much of the research on turnover among nurses so far focuses predominantly on predictors of nurses’ intention to leave their current job or organisation or, to a lesser extent, their current profession [4–8]. Little attention has been paid to factors related to their self-perceived ability to continue working in the current line of work until retirement age [9]. As far as we know, to date no model has been developed to explain these factors.

Karsh et al. [10] created a general model of nursing staff turnover that integrates elements from different models examined by Price and Mueller [11], Hinshaw et al. [12] and Parasuraman (as cited in [10]) and a meta-analysis conducted by Irvine and Evans [13]. According to this model, job satisfaction and organisational commitment are the key factors determining turnover intention. Several studies have identified positive associations between job satisfaction and organisational commitment on the one hand and turnover intention on the other hand (e.g. [14–16]). Furthermore, the model of Karsh*,* et al. [10] showed that various job, organisational, economic and demographic factors in turn affect both job satisfaction and organisational commitment. None of these different types of factors have a direct effect on turnover intention.

Multiple literature reviews and meta-analyses have identified the following job and organisational factors as affecting job satisfaction and/or organisational commitment: autonomy, workload and stress, leadership style and educational opportunities [13, 17–22]. Autonomy and educational opportunities had positive associations, while the associations with workload and stress were negative. In their systematic review of leadership styles and outcome patterns for the nursing workforce, Cummings et al. [23] found that leadership styles focused on people and relationships were positively related to nurse job satisfaction, while leadership styles focused on tasks were negatively related to nurse job satisfaction. Furthermore, earlier research suggests that greater communication within the organisation and perceived appreciation by senior management are also positively related to job satisfaction and/or organisational commitment. There are multiple studies in which nursing staff indicate that increased appreciation in the organisation for their work would make the profession more appealing [24, 25]. In addition, Liou [26] argued that communication between administrators and nursing staff is vital to generate the level of organisational commitment that is needed for a durable, effective work environment.

The aim of our study was to identify job and organisational factors related to the self-perceived ability of nursing staff to continue in their current line of work until the official retirement age, using the model of Karsh*,* et al. [10] as a starting point. This general model of nursing staff turnover might also be applicable to the self-perceived ability of nursing staff to remain working until retirement. In our study, we focused on job factors and organisational factors, as these factors can be influenced by employers. Since our study examined the self-perceived ability to continue working in the current line of work rather than the current job, occupational commitment was included instead of organisational commitment. Occupational commitment has been found to have a stronger link to occupational turnover intention than organisational commitment [27].

## Meth**o**ds

### Hypothesised associations

**Fig. 1:**
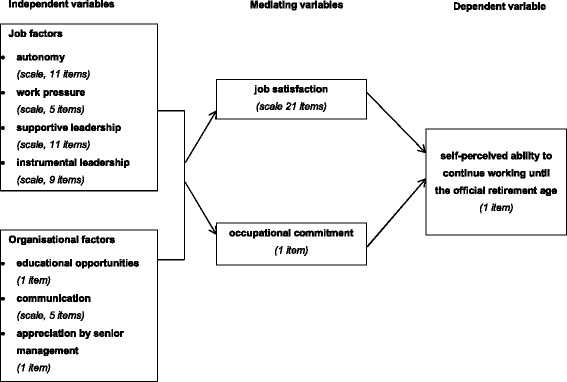
Associations examined in this study

Figure 1 shows the hypothesised associations examined in our study.

We tested the following hypotheses:

1. Job factors (i.e. more autonomy, less work pressure, more supportive leadership and less instrumental leadership) and organisational factors (i.e. more educational opportunities, better communication and more appreciation of nursing staff by senior management) are positively related to job satisfaction.
2. These job factors and organisational factors are also positively related to occupational commitment.
3. Nursing staff members who are less satisfied with their work or less committed to their occupation are more likely to think that they will be unable to continue working in the current line of work until the official retirement age.
4. The relationships between job factors and organisational factors on the one hand and the self-perceived ability of nursing staff to continue in their current line of work until the official retirement age on the other hand are mediated by job satisfaction and occupational commitment.

### Design and setting

Our hypotheses were evaluated using a cross-sectional correlational study, based on a secondary analysis of two datasets:

1. a questionnaire survey containing questions about the self-perceived ability to continue working, with data collection in January 2011 (75 % response rate)
2. a questionnaire survey containing questions about job satisfaction, occupational commitment, job factors and organisational factors, with data collection in May 2011 (68 % response rate)

The analysis is termed’secondary analysis’ as the data on job satisfaction, occupational commitment, job factors and organisational factors were not originally gathered for the purpose of explaining the self-perceived ability to continue working. Both questionnaires were self-administered. Respondents could complete the first questionnaire online or on paper. The second questionnaire was sent by post and completed on paper. The data from the two questionnaires were linked using the unique respondent ID.

Our research was conducted in the Netherlands. Hence, we used the Dutch official retirement age, which was 65 for both men and women at the time.

### Sample

A total of 730 Dutch nursing staff members completed both questionnaires. All respondents were members of a pre-existent research sample, the Nursing Staff Panel, consisting of a nationally representative group of registered nurses, certified nursing assistants and social workers in Dutch hospitals, nursing homes, organisations for psychiatric care, homes for the elderly, care organisations for disabled people and home-care organisations. They deliver direct patient care and are willing to fill in questionnaires about current topics in health care. Candidates for the panel are recruited from a random sample of employees in healthcare organisations.

The vast majority of respondents (89 %) were female (Table 1). The respondents’ mean age of 47 (standard deviation, or *S.D.* = 9.3) was several years older than the mean age of the Dutch population of nursing staff. Most respondents (88 %) only delivered direct patient care, while 12 % also had managerial tasks. The respondents were employed for 26 h a week on average (*S.D.* = 7.5). A large proportion of the respondents (74 %) had irregular shifts. Their average score for self-perceived health was 4.1 (*S.D.* = 0.6) on a five-point scale ranging from 1 = ‘very poor’ to 5 = ‘very good’.

**Table 1:** Descriptive statistics for variables in analyses: means, missing data, scale reliabilities (*N* = 730)

	% or mean (S.D.)	Missing data (%)	Cronbach’s α^a^
Dependent variable			
Self-perceived ability to continue working		0.3 %	N/A
No	43.4 %		
Yes or don’t know	56.6 %		
Mediator variables			
Job satisfaction (range 1–5)	3.59 (0.48)	1.6 %	0.91
Occupational commitment (range 1–5)	4.05 (0.86)	1.2 %	N/A
Independent variables			
* Job factors*			
Autonomy (range 1–4)	2.70 (0.54)	0.4 %	0.91
Work pressure (range 1–5)	2.90 (0.80)	0.8 %	0.84
Supportive leadership (range 1–5)	3.53 (0.73)	3.4 %	0.91
Instrumental leadership (range 1–5)	2.82 (0.59)	3.8 %	0.82
*Organisational factors*			
Sufficient educational opportunities		1.5 %	N/A
No	31.6 %		
Yes	68.4 %		
Communication (range 1–4)	2.52 (0.61)	1.2 %	0.87
Appreciation by senior management (range 1–4)	2.47 (0.72)	1.4 %	N/A
Respondent characteristics (control variables)			
Age (years)	46.79 (9.3)	0.0 %	N/A
Gender		0.0 %	N/A
Male	10.8 %		
Female	89.2 %		
Perceived health (range 1–5)	4.11 (0.61)	3.3 %	N/A
Working hours per week	25.58 (7.48)	2.7 %	N/A
Irregular shifts		2.9 %	N/A
No	26.2 %		
Yes	73.8 %		
Managerial tasks		0.3 %	N/A
No	88.0 %		
Yes	12.0 %		

^a^N/A = not applicable

### Measures

#### Self-perceived ability to continue working until the age of 65

The self-perceived ability to continue working until the official retirement age was assessed using the question ‘Do you think you are able to continue working in your current line of work until the age of 65?’ , which was used in a large-scale national study of work circumstances [28]. The responses were originally on a three-point scale (‘yes’ , ‘no’ and ‘don’t know’) and subsequently dichotomised. Respondents answering ‘yes’ or ‘don’t know’ (score = 1) were classified as (probably) being able to continue in their current line of work or being unsure whether they could continue, whereas respondents answering ‘no’ were classified as not being able to continue in their current line of work (score = 0). This classification was applied since this study focused on those nursing staff members who believe they are not able to continue working until retirement and factors that could change this conviction.

#### Job satisfaction

Job satisfaction was measured using the shortened version of the MAS-GZ (Maastricht Work Satisfaction Scale for Healthcare) by Landeweerd et al. [29]. This instrument comprises 21 items covering seven dimensions of job satisfaction: ‘supervisor’ , ‘quality of care’ , ‘contacts with colleagues’ , ‘contacts with patients’ , ‘possibilities for promotion’ , ‘opportunities for self-actualisation/growth’ and ‘clarity of tasks and rules’. Each item was rated on a five-point Likert scale ranging from 1 = ‘very dissatisfied’ to 5 = ‘very satisfied’. Overall job satisfaction was calculated as the mean score of all 21 items (Cronbach’s alpha = 0.91).

#### Occupational commitment

Occupational commitment was operationalised using the statement ‘I’m proud to be in the nursing profession’ (Additional file 1). Respondents could indicate whether they agreed with this statement on a five-point Likert scale ranging from 1 = ‘strongly disagree’ to 5 = ‘strongly agree’.

#### Autonomy

Self-perceived professional autonomy was measured using the ‘autonomy’ subscale (Cronbach’s alpha = 0.91) from the ‘Experience and Assessment of Work’ (VBBA) questionnaire by Van Veldhoven and Meijman [30]. Some examples of the 11 items are ‘Do you have freedom in carrying out your daily activities? ’ and ‘Do you solve problems yourself? ’. The responses were on a four-point Likert scale ranging from 1 = ‘never’ to 4 = ‘always’.

#### Work pressure

The work pressure experienced was measured using a five-item scale (Cronbach’s alpha = 0.84), giving an assessment of the time available for direct patient care [31]. The items were rated on a five-point Likert scale ranging from 1 = ‘I fully agree’ to 5 = ‘I fully disagree’. One example of these items is ‘I have enough time to give good care to patients’.

#### Leadership style

Leadership style was assessed with the Leader Behaviour Description Questionnaire (Stogdill [32], revised and translated by Boumans [33]). This instrument consists of two subscales, measuring different dimensions of leadership: supportive leadership and instrumental leadership. Supportive leadership behaviour focuses on the personal needs of employees, whereas instrumental leadership behaviour is goal oriented and focuses on completing tasks. The two subscales (Cronbach’s alpha = 0.91 and 0.82) consisted of eleven and nine items. Respondents could indicate how often their manager showed such behaviour using a five-point Likert scale from 1 = ‘never’ to 5 = ‘always’.

#### Educational opportunities

Educational opportunities were measured by asking whether the respondent approves of the amount of personnel training (0 = ‘no’ , 1 = ‘yes’) (Additional file 1).

#### Communication

Communication within the organisation was assessed using an adapted version of the ‘communication’ subscale from the ‘Experience and Assessment of Work’ (VBBA) questionnaire by Van Veldhoven and Meijman [30]. Some examples of the items are ‘Are you kept adequately up to date about important issues within the organisation? ’ and ‘Is it clear to you whom you should address within the organisation for specific problems? ’ One self-developed item was added: ‘Are you able to give your opinion on important policy decisions to the senior management?’. The scale contained five items and responses were on a four-point Likert scale ranging from 1 = ‘never’ to 4 = ‘always’. Cronbach’s alpha is 0.87.

#### Appreciation by senior management

Nursing staff's perceived appreciation by senior management was operationalised with the following question: ‘Do you feel appreciated by the senior management within the organisation? ’ (Additional file 1). Respondents could indicate their answer on a four-point Likert scale ranging from 1 = ‘not at all’ to 4 = ‘to a large extent’.

### Ethical considerations

This study was questionnaire based and had no patient involvement. According to Dutch law (www.ccmo.nl), no ethical approval was needed because the research subjects were not subjected to any interventions or actions. Study participation was voluntary and anonymous.

### Data analysis

Since the hypothesised associations can be influenced by the individual characteristics of the nursing staff, we controlled for age, gender, self-perceived health, number of working hours per week, working irregular shifts, performing managerial tasks and educational level.

Linear regression analysis was used to test hypotheses 1 and 2. In the first step simple linear regression analyses were used to select the independent variables for use in a multiple regression model. Separate linear regression analyses were conducted with one independent variable (a job factor, organisational factor or respondent characteristic) and one dependent variable (job satisfaction or occupational commitment). Because of the large number of tests, multiplicity adjustment was necessary; this was accomplished by using 99 % confidence intervals. Next, the job factors, organisational factors and respondent characteristics with *P* < 0.01 in the univariate regression analysis were selected as independent variables for inclusion in the multiple linear regression analysis. Two multiple regression analyses were performed; one with job satisfaction as the dependent variable and one with occupational commitment as the dependent variable.

Logistic regression analysis was used to test hypotheses 3 and 4. In the first step simple logistic regression analyses were used to select the independent variables for use in a multiple logistic regression model. Separate logistic regression analyses were performed with one independent variable (job satisfaction, occupational commitment, one of the job factors, one of the organisational factors or one of the respondent characteristics). The ability to continue working was the dependent variable. Because of multiple testing, a 99 % confidence interval was applied to adjust for multiplicity. Second, multiple logistic regression analysis was used to predict the ability to continue working based on both job satisfaction and occupational commitment, while controlling for the influence of the respondent characteristics (hypothesis 3). In this analysis, job satisfaction, occupational commitment and respondent characteristic(s) with *P* < 0.01 in the univariate logistic regression analysis were included as independent variables and the ability to continue working was included as the dependent variable. A check was also made for possible direct relationships between job factors and organisational factors on the one hand and the ability to continue working on the other hand using a second multiple logistic regression analysis, with the ability to continue working as the dependent variable. The independent variables in this analysis were job satisfaction and occupational commitment, plus the job factors, organisational factors and respondent characteristics with *P* < 0.01 in the univariate logistic regression analyses. To assess the mediated effect of job satisfaction and occupational commitment (hypothesis 4), mediation analysis was conducted using logistic regression analysis with standardised coefficients [34, 35]. Separate mediation analyses were performed with the self-perceived ability to continue working as the dependent variable, job satisfaction or occupational commitment as the mediator and one of the job factors or organisational factors that were significant in the multiple linear regression analyses as the independent variable. Standard errors for the direct and indirect effects along with 95 % confidence intervals were obtained by bootstrapping (500 replications).

The data management and analysis were performed using STATA 12.1 (2011). Respondents with missing values for one or more variables were excluded from the analyses containing those variables. Table 1 shows the proportion of missing data for each variable. The assumptions on which multiple linear and logistic regression analyses are based were checked (i.e. the absence of multicollinearity, homoscedasticity, the linearity of the dependent variable, linear relationships between predictor variables and the outcome (or its log), normally distributed residual terms and absence of dispersion). Job satisfaction and occupational commitment showed a deviation from linearity. Therefore robust multiple linear regression was also conducted. The results did not diverge substantially from the initial multiple regression analyses. No further violations of assumptions were found.

## Results

### Self-perceived ability to continue working

As can be seen from the data in Table 1, 43 % of the respondents did not think they would be able to continue working in their current line of work until the age of 65. The proportion of respondents who thought they would be able to continue working, or were not sure about this, was 57 %.

### Job factors and organisational factors related to job satisfaction

Bivariate analyses showed that all job and organisational factors measured were indeed related to job satisfaction (Table 2). However, instrumental leadership showed no association with job satisfaction when other job and organisational factors were included in the analysis (Table 3). So this largely confirms our first hypothesis, that job satisfaction is related to the selected job and organisational factors. Interestingly, the job and organisational factors explained a large proportion of the variance in job satisfaction (62 %). Hence, these factors are important in explaining nursing staff’s satisfaction with their job.

**Table 2:** Simple regression predicting ‘job satisfaction’, ‘occupational commitment’ and ‘self-perceived ability to continue working’

	Job satisfaction	Occupational commitment	Self-perceived ability to continue working
	Coefficient	Coefficient	Coefficient
	Simple linear regression (99 % C.I.)	Simple linear regression (99 % C.I.)	Simple logistic regression (99 % C.I.)
	*N* = 695–718	*N* = 696–721	*N* = 700–728
Mediators			
Job satisfaction (range 1–5)	-	-	0.77 (0.35 – 1.21)**
Occupational commitment (range 1–5)	-	-	0.25 (0.02 – 0.48)**
Job factors			
Autonomy (range 1–4)	0.40 (0.32 – 0.48)**	0.34 (0.19 – 0.49)**	0.48 (0.11 – 0.85)**
Work pressure (range 1–5) ^#^	−0.32 (−0.37 – -0.27)**	−0.27 (−0.37 – -0.17)**	−0.46 (−0.71 – -0.21)**
Supportive leadership (range 1–5)	0.43 (0.39 – 0.48)**	0.25 (0.14 – 0.37)**	0.26 (−0.01 – 0.53)
Instrumental leadership (range 1–5)^#^	−0.12 (−0.20 – -0.05)**	−0.14 (−0.28 – 0.00)	−0.19 (−0.52 – 0.14)
Organisational factors			
Educational opportunities (no = ref.)	0.35 (0.25 – 0.44)**	0.10 (−0.08 – 0.28)	0.17 (−0.24 – 0.59)
Communication (range 1–4)	0.39 (0.33 – 0.46)**	0.33 (0.20 – 0.46)**	0.39 (0.06 – 0.71)**
Appreciation by senior management (range 1–4)	0.32 (0.26 – 0.37)**	0.31 (0.20 – 0.42)**	0.49 (0.21 – 0.77)**
Respondent characteristics			
Age	−0.00 (−0.01 – 0.00)	−0.00 (−0.01 – 0.01)	0.03 (0.01 – 0.05)**
Gender (woman = ref.)	−0.10(−0.24 – 0.05)	−0.16 (−0.42 – 0.11)	0.48 (−0.17 – 1.13)
Perceived health (range 1–5)	0.09 (0.01 – 0.17)**	0.15 (0.01 – 0.28)**	0.47 (0.14 – 0.80)**
Working hours per week	−0.01 (−0.01 – 0.00)	−0.00 (−0.01 – 0.01)	0.02 (−0.01 – 0.04)
Irregular shifts (no = ref.)	−0.05 (−0.16 – 0.05)	−0.08 (−0.27 – 0.11)	−0.24 (−0.68 – 0.21)
Managerial tasks (no = ref.)	0.09 (−0.05 – 0.24)	0.15 (−0.10 – 0.41)	−0.01 (−0.61 – 0.58)

-Variable is not included in the analysis

^#^Scale is in opposite direction; negative relationships are expected

**Statistically significant with *p* < 0.01

**Table 3:** Multiple regression predicting ‘job satisfaction’ , ‘occupational commitment’ and ‘self-perceived ability to continue working’

	Job satisfaction	Occupational commitment	Self-perceived ability to continue working	
	Coefficient	Coefficient	Coefficient	
	Multiple linear regression (95 % C.I.)	Multiple linear regression (95 % C.I.)	1) Multiple logistic regression (95 % C.I.)	2) Multiple logistic regression (95 % C.I.)
	*N* = 660	*N* = 667	*N* = 688	*N* = 673
Mediators				
Job satisfaction (range 1–5)	-	-	0.72 (0.36 – 1.08)**	0.26 (−0.21 – 0.74)
Occupational commitment (range 1–5)	-	-	0.09 (−0.11 – 0.29)	0.07 (−0.13 – 0.27)
Job factors				
Autonomy (range 1–4)	0.13 (0.08 – 0.18)**	0.12 (−0.01 – 0.25)	-	0.09 (−0.25 – 0.43)
Work pressure (range 1–5)^#^	−0.15 (−0.18 – -0.12)**	−0.16 (−0.24 – -0.07)**	-	−0.30 (−0.54 – -0.06)*
Supportive leadership (range 1–5)	0.28 (0.25 – 0.32)**	0.06 (−0.03 – 0.16)	-	
Instrumental leadership (range 1–5)^#^	−0.00 (−0.04 – 0.04)	-	-	
Organisational factors				
Educational opportunities (no = ref.)	0.12 (0.07 – 0.17)**	-	-	
Communication (range 1–4)	0.06 (0.01 – 0.11)*	0.06 (−0.08 – 0.19)	-	−0.13 (−0.47 – 0.21)
Appreciation by senior management (range 1–4)	0.07 (0.03 – 0.11)**	0.17 (0.07 – 0.28)**	-	0.34 (0.05 – 0.62)*
Respondent characteristics				
Age	-	-	0.04 (0.02 – 0.05)**	0.04 (0.02 – 0.05)**
Gender (woman = ref.)	-	-	-	
Perceived health (range 1–5)	0.04 (−0.00 – 0.07)	0.11 (0.01 – 0.22)*	0.47 (0.20 – 0.74)**	0.44 (0.17 – 0.71)**
Working hours per week	-	-	-	
Irregular shifts (no = ref.)	-	-	-	
Managerial tasks (no = ref.)	-	-	-	
Test of model	*R* ^*2*^ = 0.62, *F* (8,651) = 137.01**	R^2^ = 0.12, F (6,660) = 14.94**	R^2^ = 0.10 (Nagelkerke), Model *X* ^*2*^(4) = 51.10**	(R^2^ = 0.12 (Nagelkerke), Model *X* ^*2*^(8) = 62.49**

-Variable is not included in the analysis

^#^Scale is in opposite direction; negative relationships are expected

*Statistically significant with *p* < 0.05

**Statistically significant with *p* < 0.01

### Job factors and organisational factors related to occupational commitment

Bivariate relationships were found between occupational commitment and five job/organisational factors: autonomy, work pressure, supportive leadership, communication and appreciation by senior management (Table 2). However, when all these factors were included in the analysis (Table 3), occupational commitment was only related to work pressure and appreciation (and the control variable self-perceived health). Those staff members who experienced less work pressure and more appreciation by senior management felt more occupational commitment. Hence, the hypothesis that occupational commitment is related to the selected job and organisational factors is only partially confirmed.

### Job satisfaction, occupational commitment and the self-perceived ability to continue working

Bivariate analyses indicated that job satisfaction and occupational commitment were associated with the self-perceived ability to continue working (Table 2). Yet, occupational commitment turned out not to be related to the ability to continue working when job satisfaction was included in the analysis (Table 3). Therefore, hypothesis 3 is only true for job satisfaction. Also, the control variables of age and self-perceived health appeared to be positively associated with the ability to continue working.

### Job satisfaction as mediator

The separate mediation models showed that job satisfaction indeed mediates the relationship between autonomy, work pressure, supportive leadership, educational opportunities, communication and appreciation by senior management on the one hand and the ability to continue working on the other hand (Table 4). Concerning educational opportunities, the mediation analysis showed somewhat surprising results at first sight. The total effect of educational opportunities was not significant, while the indirect effect was significant. In this mediation analysis, the direct effect was opposite in sign to the indirect effect, and so the job satisfaction mediator could have acted as a suppressor variable. In such a case there is still mediation [35]. Contrary to expectations, work pressure and appreciation by senior management also showed a direct relationship with the ability to continue working in addition to the indirect relationship through job satisfaction (Table 3 and Table 4). Multiple regression analysis comparing nested models showed no cumulative effect of the other job factors and organisational factors on the ability to continue working. Due to the correlations with these factors, job satisfaction no longer predicted the ability to continue working when job factors and organisational factors were included in the multiple logistic regression analysis (Table 3). Job satisfaction does not contribute separately to the prediction of the ability to continue working when account is taken of the joint contributions of the job factors and organisational factors. Job satisfaction seems to encapsulate the individual effects of the different job factors and organisational factors, without having a supplementary effect on the self-perceived ability to continue working. Thus, job satisfaction mediates the separate relationships between job factors and organisational factors (except instrumental leadership) on the one hand and the self-perceived ability to continue working on the other hand, but has no mediating role when linking these factors simultaneously to the self-perceived ability to continue working until retirement. No mediation analyses were performed for occupational commitment since it was not related to the self-perceived ability to continue working when account was taken of job satisfaction. In conclusion, hypothesis 4 is rejected.

**Table 4:** Separate mediation analyses with ‘job satisfaction’ as the mediating variable and ‘self-perceived ability to continue working’ as the dependent variable ^a^ ^b^

Independent variables	*N*	Total effect	Total indirect effect	Direct effect
		Coefficient (95 % C.I.)	Coefficient (95 % C.I.)	Coefficient (95 % C.I.)
Job factors				
Autonomy (range 1–4)	715	0.140 (0.056 – 0.233)*	0.077 (0.037 – 0.125)*	0.063 (−0.034 – 0.160)
Work pressure (range 1–5)^#^	712	−0.197 (−0.272 – -0.117)*	−0.070 (−0.127 – -0.016)*	−0.127 (−0.219 – -0.031)*
Supportive leadership (range 1–5)	702	0.106 (0.026 – 0.188)*	0.162 (0.091 – 0.244)*	−0.056 (−0.175 – 0.060)
Organisational factors				
Educational opportunities (no = ref.)	705	0.043 (−0.050 – 0.124)	0.070 (0.040 – 0.108)*	−0.026 (−0.119 – 0.063)
Communication (range 1–4)	710	0.134 (0.052 – 0.227)*	0.087 (0.035 – 0.139)*	0.047 (−0.043 – 0.149)
Appreciation by senior management (range 1–4)	709	0.198 (0.114 – 0.286)*	0.070 (0.027 – 0.119)*	0.128 (0.032 – 0.224)*

^a^Mediation effects estimated by bootstrapping (500 replications)

^b^Percentile confidence intervals (no bias correction)

^#^Scale is in opposite direction; negative relationships are expected

*Statistically significant with *p* < 0.05

## Discussion

### Main findings

The present study was designed to gain insight into the associations between different job and organisational characteristics, job satisfaction, occupational commitment and the self-perceived ability of nursing staff to continue working in the current line of work until the official retirement age. Our results showed that work pressure and appreciation by senior management in particular are important in explaining the self-perceived ability to continue working in the current line of work. The job characteristics of autonomy, work pressure and supportive leadership and the organisational characteristics of educational opportunities, communication and appreciation by senior management together have substantial impact on job satisfaction. Furthermore, only work pressure and appreciation are significantly related to occupational commitment when accounting for the effect of the other job and organisational characteristics. Job satisfaction is related to the self-perceived ability to continue working, whereas occupational commitment has no contribution over and above job satisfaction in explaining this self-perceived ability. However, job satisfaction mainly summarises the joint effect of job and organisational factors and has no separate, supplementary effect on the self-perceived ability to continue working.

The results of this study confirm previous findings (e.g. [13] [18] [21]) and provide additional evidence of the associations between job satisfaction and the job and organisational factors of autonomy, work pressure, leadership style and educational opportunities. Nursing staff members who are allowed to have a certain degree of self-determination, freedom and discretion over their job, who have sufficient time to deliver high-quality care to patients, whose supervisor focuses on their personal needs and who have opportunities for professional development are more satisfied with their job.

Also, the current study showed that perceived appreciation in the organisation for nursing staff’s work not only makes the profession more appealing [24], it seems also to be positively related to job satisfaction. The finding that communication within the organisation is associated with job satisfaction elaborates upon the ideas of Liou [26], who argued that communication between administrators and nursing staff is important in achieving a durable, effective work environment.

Our study showed an association between job satisfaction and the self-perceived ability to continue in the current line of work until the official retirement age. Thus, nursing staff members who are highly satisfied with their job are not only less likely to resign from their job or leave the profession, as earlier studies described (e.g. [6, 14, 16]), they are also less likely to believe they are unable to remain working in the current line of work until they reach retirement age.

According to this study, occupational commitment is not related to the self-perceived ability to continue working once account has been taken of the influence of job satisfaction. Previous studies have reported an association between occupational or professional commitment and nurses’ intention to leave the profession [6]. However, job satisfaction seems to outweigh occupational commitment when it comes to staying in the profession until retirement age.

Our study also indicates that the only job and organisational factors with a direct effect on the self-perceived ability to continue working are work pressure and appreciation of nursing staff by senior management. This emphasises the negative consequences of high work pressure and a lack of appreciation. Limiting nursing staff’s work pressure and showing appreciation for their work seem to be vital if employers are to retain them.

In line with the literature [9, 36], our study indicates that age and self-perceived health are important in explaining the self-perceived ability to continue working until retirement age, in addition to the aforementioned job and organisational factors. Also, other individual factors (e.g. family needs) may play a role. However, although good health and other personal factors are important in prolonging working life, these factors are part of nursing staff members’ personal context and predominantly beyond the reach of employers. However, employers could boost the personal health of their employees, for example by initiating a vitality programme and promoting healthy working conditions.

### Strengths and limitations

The current findings make several contributions to the literature. First, while a large body of literature has been devoted to the issue of nursing staff leaving the current job, organisation or profession, little research has been conducted on continuing working in the current line of work until retirement [9]. Furthermore, in contrast to the substantial amount of research on the working conditions of nursing staff within a particular healthcare sector [4, 8], the participants in this study encompassed nursing staff in different healthcare sectors. Finally, our study adds to previous studies on the relationship between job and organisational characteristics and job satisfaction (e.g. [13, 17]), by showing that communication within the organisation and perceived appreciation by senior management are also associated with job satisfaction.

Our study has some limitations. First, we assume that the self-perceived ability to remain working in the current line of work predicts whether nursing staff will in fact continue working until retirement age. Yet, this cannot be verified with our data because we did not measure how long nurses actually continued working. However, if nursing staff members think they are unable to continue in the current line of work until the age of 65, it seems plausible that they will retire early or make a career change. Ybema et al. [37] found the self-perceived ability to continue working until the age of 65 to be predictive of early retirement in the general working population aged 45 to 64. Another limitation is that the cross-sectional design of our study means that no cause-and-effect conclusions can be drawn. However, it is unlikely that the self-perceived ability to continue working in the current line of work precedes job and organisational factors such as autonomy and supportive leadership. Furthermore, selection bias could have affected the study results. However, the high response rate for both questionnaires suggests that this was probably not the case. Finally, we used a single item measure for occupational commitment rather than a scale. Using a scale could have enhanced the reliability of this measure.

### Suggestions for future work

Additional empirical research could further validate the associations found. Longitudinal studies are recommended to ascertain the cause-and-effect relationships between job and organisational factors on the one hand and the self-perceived ability to continue working on the other hand. Preferably, these longitudinal studies should include a measurement of whether nursing staff members actually continue working until the official retirement age. This would enable the prognostic value of the self-perceived ability to continue working to be estimated.

## Conclusions

To maintain a balanced nursing labour market, it is crucial to understand the job characteristics and organisational characteristics that are important in retaining nurses in patient care. With this knowledge, employers can focus on the most essential working conditions. This study has shown that employers should primarily address work pressure and appreciation of nursing staff by senior management as this would have a positive effect on nursing staff’s ability to remain working until retirement. Furthermore, this study has revealed that employers can keep nursing staff members satisfied with their job by encouraging autonomy in nursing practice, regulating the work load, motivating team leaders to listen to nursing staff members and support them when they need help, fostering educational opportunities, advocating communication between administrators and nursing staff, and encouraging people in senior management positions to show their appreciation of nursing staff. Aiken et al. [38] reported 11 % (Netherlands) to 56 % (Greece) of nurses in general acute care hospitals in twelve European countries to be dissatisfied with their job, with an average of 30 %. Therefore, it seems crucial for healthcare organisations to tackle the sources of job dissatisfaction among nursing staff.

## Additional file

Additional file 1:
**Questionnaire items.** (DOCX 16 kb)
