# Supplementary material for: Factors associated with the self-perceived ability of nursing staff to remain working until retirement: a questionnaire survey
Source: BMC Health Serv Res. 2015 Sep 2;15:356. doi: 10.1186/s12913-015-1006-x (PMC4557922; doi:10.1186/s12913-015-1006-x)
Supplement: Additional file 1: — Questionnaire items. (DOCX 16 kb) [file 12913_2015_1006_MOESM1_ESM.docx]

**Questionnaire items**

The following list of questionnaire items contains the items that were developed by the authors. These items were originally in Dutch and translated into English for the purpose of writing this paper.

In case of use please refer to our paper (Maurits EEM, De Veer AJE, Van der Hoek LS, Francke AL: Factors associated with the self-perceived ability of nursing staff to remain working until retirement: A questionnaire survey. *BMC Health Services Research* 2015).

For access to items and scales developed by others, please use the references cited in our paper.

*Occupational commitment*

Statement Response options

I’m proud to be in the nursing profession’ 1 = ‘strongly disagree’

2 = ‘disagree’

3 = ‘neither agree nor disagree’

4 = ‘agree’

5 = ‘strongly agree’

*Educational opportunities*

Question Response options

Do you approve the amount of personnel 0 = ‘no

training provided by your employer? 1 = ‘yes’

*Appreciation by senior management*

Question Response options

Do you feel appreciated by the senior 1 = not at all

management within the organisation? 2 = little

3 = enough

4 = to a large extent
